# Zinc Complexes with 1,3-Diketones as Activators for Sulfur Vulcanization of Styrene-Butadiene Elastomer Filled with Carbon Black

**DOI:** 10.3390/ma14143804

**Published:** 2021-07-07

**Authors:** Magdalena Maciejewska, Anna Sowińska, Agata Grocholewicz

**Affiliations:** Department of Chemistry, Institute of Polymer and Dye Technology, Lodz University of Technology, Stefanowskiego Street 12/16, 90-924 Lodz, Poland; 233551@edu.p.lodz.pl

**Keywords:** zinc complexes, 1,3-diketones, vulcanization, cure characteristics, mechanical properties

## Abstract

Zinc oxide nanoparticles (N-ZnO) and zinc complexes with 1,3-diketones of different structures were applied instead of microsized zinc oxide (M-ZnO) to activate the sulfur vulcanization of styrene-butadiene rubber (SBR). The influence of vulcanization activators on the cure characteristics of rubber compounds, as well as crosslink density and functional properties of SBR vulcanizates, such as tensile properties, hardness, damping behavior, thermal stability and resistance to thermo-oxidative aging was explored. Applying N-ZnO allowed to reduce the content of zinc by 40% compared to M-ZnO without detrimental influence on the cure characteristic and performance of SBR composites. The activity of zinc complexes in vulcanization seems to strongly depend on their structure, i.e., availability of zinc to react with curatives. The lower the steric hindrance of the substituents and thus the better the availability of zinc ions, the greater was the activity of the zinc complex and consequently the higher the crosslink density of the vulcanizates. Zinc complexes had no detrimental effect on the time and temperature of SBR vulcanization. Despite lower crosslink density, most vulcanizates with zinc complexes demonstrated similar or improved functional properties in comparison with SBR containing M-ZnO. Most importantly, zinc complexes allowed the content of zinc in SBR compounds to be reduced by approximately 90% compared to M-ZnO.

## 1. Introduction

Sulfur vulcanization is the oldest and one of the most widely exploited method for the crosslinking of rubbers having unsaturated double bonds in their macromolecules, such as natural rubber (NR) and synthetic rubbers, e.g., styrene-butadiene rubber (SBR) [1]. Standard sulfur crosslinking system consists of elementary sulfur as crosslinking agent, vulcanization accelerators and activators. Additionally, many sulfur donors can be applied as curatives instead of elementary sulfur. Even though, sulfur vulcanization has been known for over 150 years, the chemistry and mechanism of sulfur crosslinking is still quite incomprehensible. It is generally accepted that when rubber compound is heated in the presence of accelerators and activators, sulfur decomposes into sulfur fragments which react with the functional groups of the rubber to create crosslinks [2]. Interaction of the accelerators and activators makes the mechanism of vulcanization more complicated due to the fact that each of reactants affects the reactivity of the other, and their interactions in turn affect the curing mechanism. Many research papers on vulcanization mechanisms have been published over the years, focusing mainly on the effect of accelerators and activators on the course of crosslinking reactions [3,4,5,6]. This confirms the important role of these ingredients in the rubber technology, since applying vulcanization accelerators and activators results in an effective and rapid crosslinking of rubber compounds. Otherwise, sulfur reacts very slowly with elastomer chains and binds to them mainly in the form of cyclic side structures, not in the form of sulfur crosslinks [2]. Accelerators and activators affect the parameters of vulcanization, such as temperature and time, as well as the safety of rubbers processing. Furthermore, they increase the crosslinks density and improve the yield of sulfur needed to form crosslinks [7]. The most widely used activator is zinc oxide (ZnO). Apart from the activation of vulcanization, ZnO has been reported to increase the crosslinking efficiency and to reduce the reversion during vulcanization. In addition, incorporation of ZnO slightly increases both the vulcanization time and the scorch time, which is crucial for the processing security [8]. Moreover, ZnO is known as a very effective and commonly used curing agent for carboxylated elastomers, leading to labile ionic crosslinks in the elastomeric network. It allows to obtain vulcanizates with high tensile strength, tear resistance, hysteresis and hardness [9]. ZnO is also a curing agent of chloroprene rubbers [10] and chlorosulfonated polyethylene [11]. Furthermore, ZnO can act as a white colorant or filler in polymer composites, especially those intended for products with increased heat conductivity [12]. Unfortunately, despite crucial role and wide applications in elastomer technology, ZnO has been classified as being very toxic to aquatic life by European Union Regulation (EC) No 1272/2008 on classification, labelling and packaging of substances and mixtures. Following the precaution recommended for ZnO in this Regulation defined as “P273: Avoid release to the environment”, the concentration of ZnO in rubber compounds, especially those used in aquatic environments, must be reduced to below 2.5 wt.%. The complete elimination of ZnO from rubber compounds is also highly recommended [13]. Hence, scientists and technologists have made efforts to reduce the amount of ZnO in rubber products or replace it with zinc compounds with a lower content of zinc ions.

The first approach to reduce the level of zinc in elastomer composites was replacing the commercially used microsized ZnO with more active forms of ZnO, which demonstrated high surface area and/or very small particle size, i.e., active ZnO or nanosized ZnO [13,14,15,16,17]. Sahoo et al. [18] applied ZnO nanoparticles with an average size of 50 nm to activate sulfur vulcanization of NR and acrylonitrile-butadiene rubber (NBR). Owing to the higher surface area and uniform dispersion of nanoparticles in the rubber matrix, the reactivity of ZnO with the curatives was improved compared to microsized ZnO, resulting in the higher crosslinking degree of the elastomer [18]. Beneficial influence of nanosized ZnO on the cure characteristics and performance of NR composites was confirmed by Panampilly et al. [19] and Roy et al. [20]. Applying N-ZnO alternatively to M-ZnO enabled to reduce the level of ZnO to 0.5 parts per hundred of rubber (phr). Thus, reducing the size of particles and increasing the specific surface area of ZnO was proven to be an effective way to decrease the level of zinc in rubber products. However, to ensure the high activity of ZnO in the vulcanization process and the required performance of the vulcanizates, it was necessary to obtain a homogeneous dispersion of nanoparticles in the elastomer matrix, which is technologically difficult due to their susceptibility to agglomeration. To overcome this disadvantage, and thus obtain a homogeneous dispersion of ZnO nanoparticles in the elastomer matrix, it is often necessary to use additional additives that act as dispersing agents of N-ZnO. The use of crown ethers [14], alkylammonium salts or ionic liquids [21] was reported to effectively improve the dispersion of N-ZnO in an elastomer matrix and, consequently, its activity in the vulcanization process. However, these additives also affect the final properties of the vulcanizates, e.g., they deteriorate the thermal stability [22,23].

Next approach to reduce the amount of zinc in rubber compounds was replacing ZnO with more chemically active zinc, in the form of reactive organic zinc complexes, in which the availability of zinc to react with curatives is better than in ZnO crystals. Hence, zinc stearate, zinc 2-ethylhexanoate and zinc m-glycerolate were employed instead of ZnO to activate the sulfur vulcanization of ethylene-propylene-diene terpolymer (EPDM) and SBR rubber [24]. Zinc m-glycerolate with the highest amount of zinc per 1 g showed the highest activity in the vulcanization. However, no reduction in the zinc amount was provided compared to rubber compounds cured with ZnO. Helaly et al. [25] applied zinc stearate to activate sulfur vulcanization of NR compounds filled with different fillers, e.g., carbon black, CaCO_3_, hydrated silicon dioxide and talc. Zinc stearate allowed for partial and complete replacement of conventional ZnO and stearic acid with improvement of the physico-mechanical properties of NR composites. However, in the case of unfilled rubber compounds, significantly lower torque increment during rheometric tests was obtained, suggesting significantly lower crosslinking degree compared to the elastomer cured with ZnO/stearic acid system. Furthermore, Maciejewska et al. [26], applied organic zinc salts and complexes, such as zinc acetylacetonate, zinc gluconate and zinc ricinoleate, as activators for sulfur vulcanization of SBR in order to reduce the level of zinc in rubber compounds. Organic zinc activators efficiently activated the vulcanization without detrimental effect on the optimal time and temperature of this process. However, due to a 70–90% lower content of zinc, SBR vulcanizates exhibited lower crosslink density compared to ZnO-containing rubber. On the other hand, reduction of the crosslink density did not worsen mechanical properties of the vulcanizates in both static and dynamic conditions. The most effective zinc activator was zinc gluconate. Moresco et al. [27] reduced the level of zinc in NR composites by 75% compared to the standard formulation with 5 phr of ZnO. Vegetable oil with aromatic zinc carboxylate were used as vulcanization activators instead of ZnO and stearic acid without affecting the mechanical performance of the elastomer composites. Moreover, the vegetable oil acted both as an activator and a lubricant of natural origin. Zachnet et al. [28] employed an activator derived from chemically modified sugar cane bagasse. It contained water, zinc carboxylate, stearic acid and lignin. The overall zinc content in this activator was 25.6%. Regarding vulcanization kinetics, this activator was a good alternative to ZnO/stearic acid system, which allowed to reduce the level of zinc by approximately 75% without detrimental influence on the crosslink density of the vulcanizates and their dynamic mechanical properties. NR composites with improved thermal stability were obtained compared to those containing 5 phr of ZnO.

The last approach to reduce the content of zinc in elastomer composites was incorporation of zinc ions into the structure of mineral layered fillers, e.g., montmorillonite (MMT) [29] and layered double hydroxides (LDHs) [30]. For example, Heideman et al. [29] developed zinc loaded clay (Zn-clay) consisted of MMT into which zinc ions were incorporated via the ion exchange reaction. Such a modified MMT was employed to activate the sulfur vulcanization of SBR alternatively to ZnO. Despite significantly lower content of zinc, Zn-clay allowed to achieve comparable curing characteristics, crosslinking degree and performance to the SBR composites cured with ZnO. Das et al. [30], prepared Zn-containing LDH filler, which delivered both zinc ions and stearate anions to activate the sulfur vulcanization of various unsaturated rubbers. The organo-modified LDH allowed to replace the ZnO and stearic acid in rubber compounds leading to significant reduction of zinc level in the final elastomer composites, without deterioration of their crosslinking degree and mechanical properties. The content of zinc was approximately 10 times lower compared to conventionally prepared composites. In addition, transparent sulfur-crosslinked elastomer composites were achieved, which is impossible when ZnO is used as a standard vulcanization activator.

To summarize, taking into account the current state of the natural environment, the topic of zinc reduction in rubber products is still very relevant. Despite the above-presented method of reducing the amount of zinc in the elastomer technology and the consequently developed vulcanization activators, it is still justified to search for new compounds that can successfully activate vulcanization while enabling the greatest possible reduction of the amount of zinc in rubber products. Thus, in this work, we applied zinc complexes with 1,3-diketones to activate the sulfur vulcanization of SBR elastomer filled with carbon black. This study is a continuation of our previous work on the use of zinc complexes to activate sulfur vulcanization of unfilled NBR compounds [31]. However, since SBR composites filled with carbon black are widely used in industry, e.g., in the automotive industry, it is justified to study the activity of zinc complexes with 1,3-diketones as vulcanization activators of such composites. Moreover, it is well known that the filler can influence the activity of the crosslinking system [32,33,34]. Therefore, the effect of filler on the efficiency of zinc complexes with 1,3-diketones as vulcanization activators should also be explored. As was already mentioned, zinc complexes with 1,3-diketones were previously applied to activate the sulfur vulcanization of unfilled NBR and the application of vulcanization activators based on organic zinc complexes alternatively to ZnO is known from the literature, but the proposed zinc complexes with aliphatic-aromatic 1,3-diketones allows to achieve the most significant reduction of the zinc level (by approximately 90%) in rubber composites compared to other organic zinc activators reported in the literature, without significantly lowering the crosslink density and most importantly with beneficial effect on the mechanical performance of the vulcanizates and their resistance to prolonged thermo-oxidation.

## 2. Materials and Methods

### 2.1. Materials

**Figure 1:**
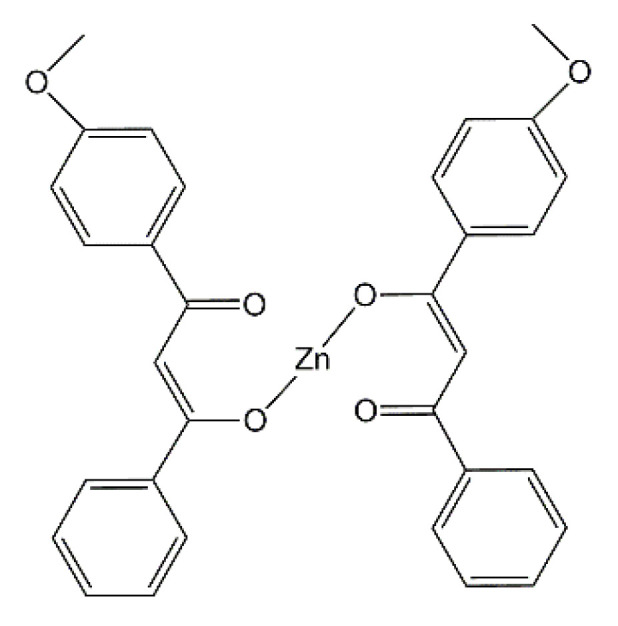
Structure of zinc 1-(4′-methoxyphenyl)-3-phenylpropane-1,3-dione (MBPP-Zn).

**Figure 2:**
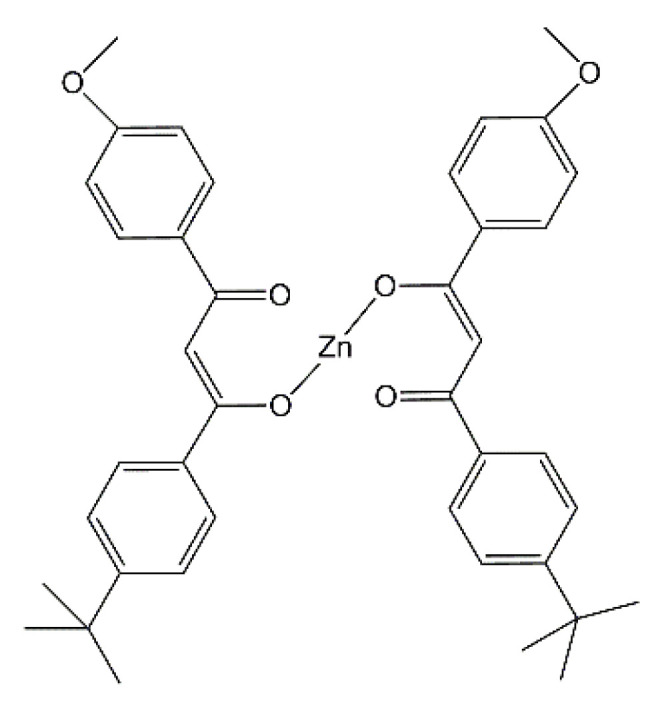
Structure of zinc 1-(4′-t-butylphenyl)-3-(4″-methoxyphenyl)propane-1,3-dione (tBuMBPP-Zn).

**Figure 3:**
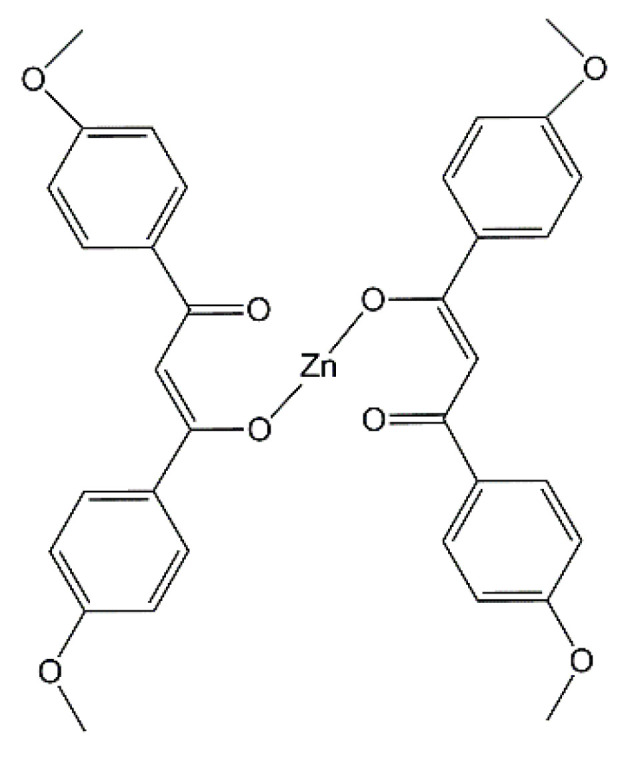
Structure of zinc 1,3-bis-(4′methoxyphenyl)propane-1,3-dione (MMBPP-Zn).

**Figure 4:**
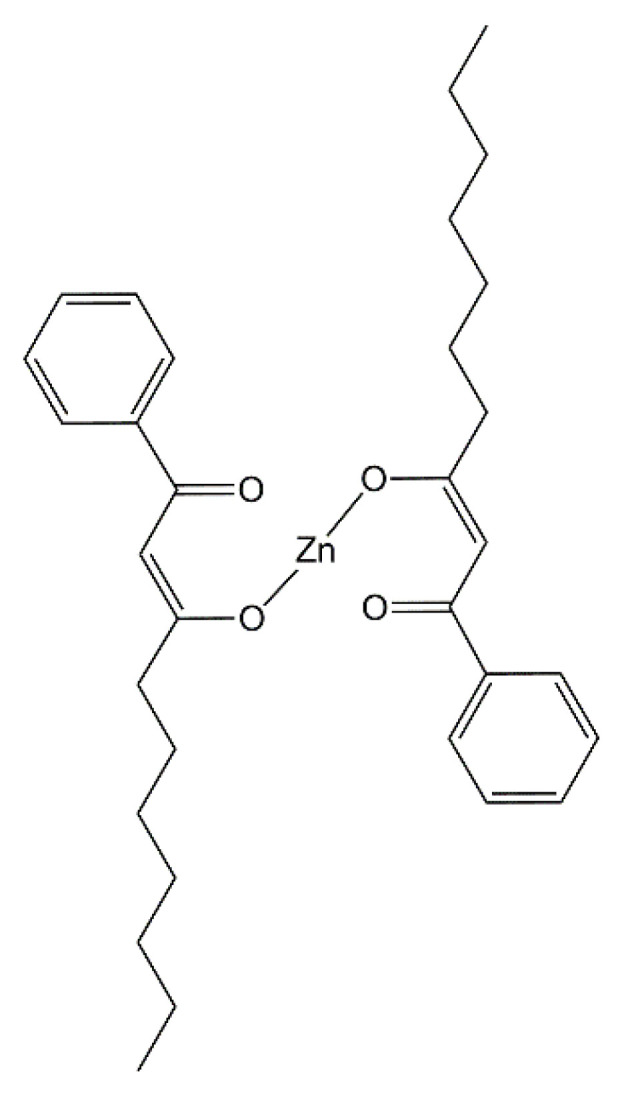
Structure of zinc 1-heptyl-3-phenylpropane-1,3-dione (HeBPP-Zn).

Styrene-butadiene rubber (SBR, KER 1500 type) was provided by Synthos SA (Oswiecim, Poland). It contains 23.5 wt.% of bonded styrene and exhibits the Mooney viscosity of ML1 + 4 (100 °C): 50. A conventional curing system containing sulfur as curing agent (Siarkopol, Tarnobrzeg, Poland) in the presence of two vulcanization accelerators, i.e., 2-mercaptobenzothiazole (MBT) and N-cyclohexyl-2-benzothiazolesulfenamide (CBS) (Sigma-Aldrich, Schelldorf, Germany) was applied for all rubber compounds. Microsized zinc oxide (M-ZnO) with a specific surface area of 10 m^2^·g^−1^ (Huta Bedzin, Bedzin, Poland) was applied as standard activator only for the reference rubber compound. Zinc oxide nanopowder (N-ZnO) with a specific surface area of 25 m^2^ g^−1^ (Sigma-Aldrich, Schelldorf, Germany) and zinc complexes with 1–3-diketones were used as alternative vulcanization activators to reduce the content of zinc in rubber compounds as compared to M-ZnO. The procedure for the synthesis of zinc complexes from commercially available 1,3-diketones has been previously published [31]. Carbon black N550 supplied by Konimpex (Konin, Poland) was used as a filler. It was characterized by the surface area of 40 m^2^·g^−1^ and pH in the range of 7–10. The structures of the zinc complexes employed as vulcanization activators are shown in Figure 1, Figure 2, Figure 3 and Figure 4.

### 2.2. Preparation and Characterization of SBR Compounds

SBR compounds were manufactured using a laboratory rolling mill (David Bridge & Co, Rochdale, UK) in a two-step procedure. The first step was to prepare the masterbatch, which consisted of the SBR rubber, curative (sulfur), accelerators (MBT and CBS) and filler carbon black (CB). The time for preparing a masterbatch including the mastication of rubber was approximately 15 min. The masterbatch was then divided into seven pieces of the same weight. Next, the proper activator (M-ZnO, N-ZnO or zinc complex, alternatively) was added to each of these pieces and mixed up for additional 5 min. The general formulations of SBR compounds are listed in Table 1 in parts per hundred of rubber (phr).

Since N-ZnO and zinc complexes were applied to reduce the content of zinc in SBR compounds, the amount of zinc ions introduced into the rubber in the form of M-ZnO, N-ZnO and particular zinc complexes with 1,3-diketones was calculated using their molecular masses and is given in Table 2. It should be noted that compared to M-ZnO, the content of zinc in SBR compounds was by approximately 40% and 90% smaller for N-ZnO and zinc complexes, respectively.

Electrically heated hydraulic press was employed to vulcanize the SBR compounds at a temperature of 160 °C and a pressure of 15 MPa. All SBR compounds were cured using the optimal vulcanization times determined during rheometric tests. The cure characteristics of SBR compounds were examined at 160 °C using the procedure described in ISO 6502 [35]. A rotorless D-RPA 3000 (MonTech, Buchen, Germany) rheometer was applied for these measurements. The optimal vulcanization time (t_90_) was established using the Equation (1), where Δ*S* is the torque increase during rheometric measurement, calculated as the difference between the maximum (*S_max_*) and minimum torque (*S_min_*), respectively. The scorch time (t_02_) was determined analogously to t_90_.

(1) $$S_{90}=0.9\Delta S+S_{min}$$

The influence of vulcanization activators on the temperature and the enthalpy of SBR compounds curing reactions was examined with a DSC1 (Mettler Toledo, Greifensee, Switzerland) differential scanning calorimeter (Mettler Toledo, Greifensee, Switzerland) equipped with a STAR^e^ software (Version 10, 2010, Mettler Toledo, Greifensee, Switzerland). The DSC measurements were carried out in the temperature range of −100–250 °C, with a heating rate of 10 °C/min. The onset curing temperature was determined following the ISO 11357-1 [36] standard using the STAR^e^ software.

Fourier transform infrared spectroscopy (FTIR) absorbance spectra were recorded in the range of wavenumbers from 4000 to 400 cm^−1^ with 64 scans. The analysis was carried out employing Thermo Scientific Nicolet 6700 (Thermo Fisher Scientific, Waltham, MA, USA) FTIR spectrometer equipped with OMNIC 8.2 software (8.2, 2010, Thermo Fisher Scientific, Waltham, MA, USA). ATR (Attenuated Total Reflectance) mode with a single reflection diamond ATR crystal was applied for all measurements.

The crosslink density of SBR vulcanizates with different vulcanization activators was determined based on solvent-swelling measurements, which were performed according to the standard ISO 1817 [37]. Four samples with a mass in the range of 20–50 mg were tested for each vulcanizate. Toluene (Chempur, Piekary Slaskie, Poland) was chosen as a solvent. Next, the Flory-Rehner equation [38] was applied to calculate the crosslink density of the vulcanizates with the Huggins parameter of SBR-toluene interaction given by Equation (2), where *V_r_* is the volume fraction of elastomer in swollen gel [39].

(2) $$\chi =0.37+0.56V_{r}$$

The mechanical properties of the vulcanizates were tested in the static and dynamic conditions. The static mechanical properties were investigated using a universal testing machine Zwick Roell 1435 (Ulm, Germany) following the procedure described in the ISO 37 [40]. Dynamic mechanical measurements were carried out in tension mode with a dynamic-mechanical analyzer DMA/SDTA861e (Mettler Toledo, Greifensee, Switzerland) analyzer. The following parameters were applied to perform DMA measurements: temperature range of −100 to 70 °C, heating rate 3 °C/min, frequency 1 Hz, strain amplitude 4 µm.

The hardness was measured by Zwick Roell 3105 (Ulm, Germany) hardness tester for disc-shaped samples of the vulcanizates according to the standard ISO 868 [41].

The standard procedure described in the ISO 188 [42] was used to perform the thermo-oxidative aging of SBR vulcanizates. Following this procedure, plates of the vulcanizates were stored in a drying chamber (Binder, Tuttlingen, Germany) at a temperature of 100 °C for 7 days. To evaluate the resistance of the vulcanizates to thermo-oxidation, their crosslink density, mechanical properties and hardness after the aging procedure were investigated and compared with the properties of non-aged samples. The aging coefficient (*AF*) was calculated according to Equation (3) [43] to quantify the resistance of SBR vulcanizates to thermo-oxidation, where *TS* is the tensile strength of vulcanizates and *EB* is the elongation at break.

(3) $$AF=\frac{{\left(EB\times TS\right)}_{after\;aging}}{{\left(EB\times TS\right)}_{before\;aging}}$$

Scanning electron microscopy (SEM) images of the vulcanizates were taken using a HITACHI S-4700 (Hitachi, Mannheim, Germany) SEM microscope. Prior to the measurements, the fractures of the vulcanizates were coated with a thin layer of carbon and next examined. Energy-dispersive-X-ray (EDS) spectroscopy was employed to investigate the dispersion of the filler and curatives (sulfur, ZnO, zinc complexes) particles in the elastomer matrix.

The effect of vulcanization activators on the thermal stability of SBR vulcanizates was explored using thermogravimetry (TG) with a TGA/DSC1 (Mettler Toledo, Greifensee, Switzerland) analyzer. First, vulcanizates were heated in the temperature range of 25–600 °C in an argon atmosphere to examine the pyrolysis of elastomer and organic additives. Then, argon was changed into air and heating was continued up to 900 °C. The heating rate used was 20 °C/min, whereas the flow rate of gases during TG measurements were 40 mL/min. The same measurement procedure was applied to study the thermal stability of pure zinc complexes with 1,3-diketones.

## 3. Results and Discussion

### 3.1. The Effect of Vulcanization Activators on the Cure Characteristics and Crosslink Density of SBR Elastomer

The content of zinc is known to affect the efficiency of activator in the vulcanization process and consequently the cure characteristics of rubber compounds [13,24]. As listed in Table 2, zinc complexes with 1,3-diketones contain significantly lower content of zinc compared to M-ZnO. Thus, they are expected to alter the vulcanization parameters of SBR compounds. The cure characteristics of SBR compounds containing different vulcanization activators are presented in Table 3.

The minimum torque (*S_min_*) during rheometric test is considered as a measure of the viscosity of the uncrosslinked rubber compound. As expected, no influence of the vulcanization activators on the *S_min_* was observed, so as expected the reduction of zinc in SBR compounds did not alter their viscosity and consequently their processing at 160 °C. Regarding the cure characteristics of rubber compounds, the torque increase (Δ*S*) during rheometric measurement is important parameter, since it corresponds to the crosslinking degree of the elastomer. The reference rubber compound with 5 phr of M-ZnO was characterized by the torque increase of 16 dNm. Despite lower content of the vulcanization activator (3 phr) and consequently the lower content of zinc, application of N-ZnO did not affect the Δ*S* of SBR compounds compared to M-ZnO. Consequently, the crosslink density of the SBR vulcanizates containing M-ZnO and N-ZnO was almost the same. Moreover, applying N-ZnO, despite lower content of zinc, did not influence the optimal vulcanization time of SBR compounds compared to M-ZnO. Thus, it was concluded that N-ZnO due to the higher specific surface area and lower particle size exhibited higher activity in the vulcanization than commercially used M-ZnO. Zinc complexes with 1,3-diketones showed lower vulcanization activity than both zinc oxides. It resulted from by approximately 80–90% smaller content of zinc compared to elastomer composites with ZnO. The most active zinc complex was HeBPP-Zn, so the one with the heptyl chain in the structure. HeBPP-Zn allowed to obtain significantly higher Δ*S* and thus, crosslink density of the vulcanizate compared to other zinc complexes, although Δ*S* of SBR compound with HeBPP-Zn was approximately 3 dNm lower compared to rubber compounds with zinc oxides. In addition, HeBPP-Zn did not significantly alter the t_90_ of SBR compounds as compared to M-ZnO and N-ZnO. SBR compounds with zinc complexes containing methoxy groups (MBPP-Zn, tBuMBPP-Zn and MMBPP-Zn) demonstrated approximately 6 dNm less Δ*S* than rubber compounds with M-ZnO due to the lower crosslinking degree of the elastomer. It was confirmed by the lower crosslink density of the vulcanizates containing these activators. Thus, zinc complexes with methoxy groups exhibited lower activity in the vulcanization than zinc oxides and HeBPP-Zn. On the other hand, SBR compounds with these zinc complexes showed approximately 50% shorter t_90_ compared to the rubber compound with M-ZnO, accompanied with the shorter scorch time (t_02_). It is worth noting that the use of a zinc complex with a methoxy group (MBPP-Zn) as a mixture (1:1) with N-ZnO improved the effectiveness of the vulcanization activator compared to pure zinc complex. As a result, SBR crosslinked with N-ZnO/MBPP-Zn as activator showed the Δ*S* and crosslink density similar to the one containing HeBPP-Zn, although still lower compared to M-ZnO or pure N-ZnO. The lower effectiveness of zinc complexes with 1,3-diketones as activators compared to M-ZnO was due to the lower content of zinc ions, which are essential for the activation of vulcanization. However, no less important than the content seems to be the availability of the zinc ions in the complex to interact with the curing system (sulfur and accelerator). Thus, despite the highest content of zinc, tBuMBPP-Zn complex in which the access to zinc ions is hindered by the presence of four phenyl rings linked to methoxy groups or t-butyl substituents (Figure 2) showed much lower activity than HeBPP-Zn (Figure 4), in which the availability of zinc ions seems to be the highest among the complexes used.

**Figure 5:**
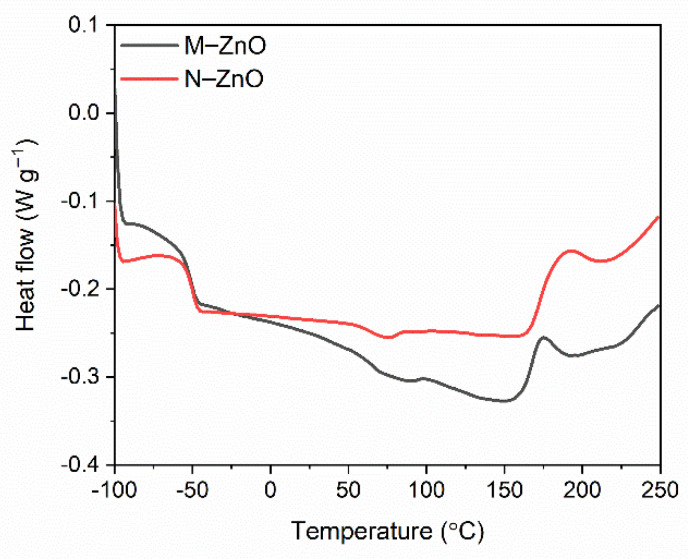
Differential Scanning Calorimetry (DSC) curves of SBR compounds containing M-ZnO and N-ZnO as activators.

**Figure 6:**
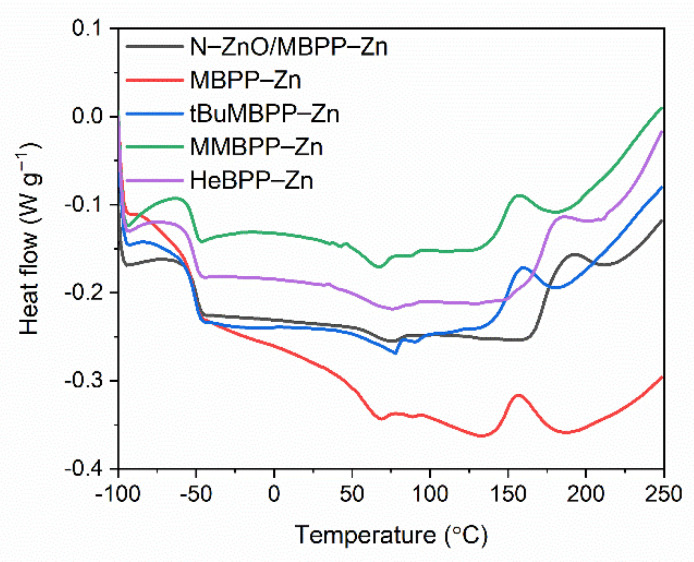
DSC curves of SBR compounds containing zinc complexes with 1,3-diketones as activators.

Having studied the influence of vulcanization activators and consequently the effect of zinc content reduction on the cure characteristics of SBR compounds, we then established the temperature and enthalpy of curing by DSC analysis. The DSC curves for SBR compounds are presented in Figure 5 and Figure 6, whereas the temperature and enthalpy of curing are summarized in Table 4.

Analyzing the differential scanning calorimetry (DSC) curves for examined SBR compounds, the first phase transition was observed as a step on the DSC curves due to the glass transition of elastomer. A midpoint of this inflection refers to the glass transition temperature (T_g_). Despite different content of zinc, SBR elastomers exhibited similar T_g_ of approximately −51 °C.

SBR compound containing M-ZnO underwent curing in a temperature range of 163–193 °C with an enthalpy of 7.2 J/g. It was a one-step process. N-ZnO did not significantly alter the temperature and enthalpy of SBR curing. Regarding zinc complexes with 1,3-diketones, HeBPP-Zn had no considerable influence on the onset curing temperature compared to SBR compound with M-ZnO, but increased the endset curing temperature from 193 to 206 °C. Thus, the SBR compound with HeBPP-Zn underwent curing in a wider temperature range than that containing M-ZnO. The enthalpy of curing for SBR with HeBPP-Zn was quite similar to the reference SBR compound with M-ZnO taking into account the measurement error. A mixture of activators, i.e., N-ZnO and MBPP-Zn, had an analogical to HeBPP-Zn impact on the curing temperature. Applying zinc complexes with methoxy groups reduced both, the onset and the endset curing temperature by approximately 20 °C compared to zinc oxides and HeBPP-Zn. In addition, SBR compounds with these zinc complexes exhibited lower enthalpies of curing as compared to other activators, so the vulcanization process seemed to be less intensive. Thus, it was concluded that despite of curing temperature reduction, zinc complexes with methoxy groups decreased the efficiency of curing leading to the formation of lower amount of crosslinks in the elastomer network, which was confirmed by the lower crosslink density of the vulcanizates containing zinc complexes with methoxy groups. It was probably due to lower content and availability of zinc ions, which is crucial for activating the vulcanization [24,25].

**Figure 7:**
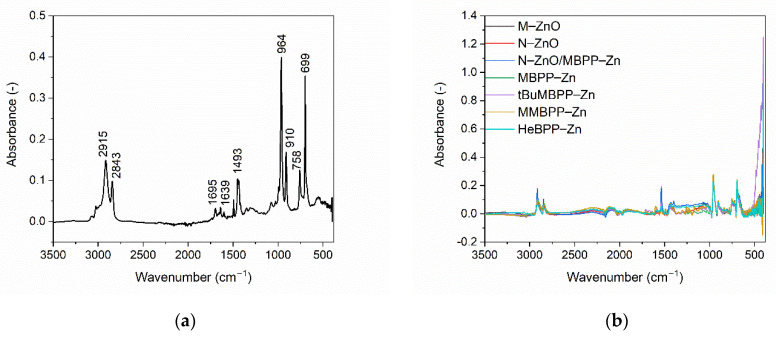
Fourier Transform Infrared (FTIR) spectra of: (**a**) pure SBR rubber; (**b**) SBR vulcanizates containing ZnO and zinc complexes with 1,3-diketones as activators.

FTIR spectroscopy was employed to study the influence of vulcanization activators on the structure of SBR vulcanizates. We intended to establish if the type of vulcanization activator, i.e., ZnO or zinc complex with 1,3-diketone, affected the structure of crosslinked elastomer network. Thus, FTIR spectra of pure SBR rubber and SBR vulcanizates were collected and presented in Figure 7.

The FTIR spectrum of the pure SBR presented in Figure 7a is consistent with the FTIR spectrum reported for this rubber by other researchers [44]. Some absorption bands corresponding to characteristic groups of SBR were observed at the following wavenumber: 2915 and 2843 cm^−1^ (stretching of C–H groups in the aromatic ring of styrene); 1695 and 1639 cm^−1^ (vibrational of stretches in CH_2_ and CH_3_ groups), and 1493 cm^−1^ (C=C aromatic). In addition, the following characteristic for SBR bands were obtained at: 699, 758, 910 and 964 cm^−1,^ which resulted from C–H out of plane deformation vibration of monosubstituted benzene, cis-1,4-butadiene and 1,2-butadiene units as well as trans-1,4-butadiene of polybutadiene chain segments, respectively [45,46].

Regarding the spectra collected for SBR vulcanizates cured with zinc oxides or zinc complexes as activators, only the bands characteristic for SBR were observed accompanied with a high intensity band at the wavenumber of approximately 490 cm^−1^, which correspond to Zn from ZnO or zinc complex. The type of vulcanization activator did not significantly affect the FTIR spectra of the vulcanizates since the spectra for the tested vulcanizates almost overlapped. Thus, it was concluded that replacing ZnO with zinc complexes did not considerably influence the structure of the crosslinked elastomer network. However, it should be mentioned that FTIR spectroscopy is not the most accurate and unambiguous method to establish the influence of the activator type on the structure of the elastomer network and the course and mechanism of the crosslinking reaction especially directly in the elastomer matrix, so using the vulcanizates. Therefore, to confirm this conclusion, other, more accurate methods should be used, such as X-ray photoelectron spectroscopy (XPS), time-of-flight secondary ion mass spectrometry (TOF-SIMS) or nuclear magnetic resonance spectroscopy (NMR), the effectiveness of which has been confirmed, for example, in the case of examining the influence of ionic liquids on the EPDM vulcanization mechanism [47]. It would also be worth conducting the research using the model compound vulcanization approach (MCV), replacing the highly viscous elastomeric medium with a liquid, low-molecular model of a fragment of the elastomer chain, e.g., 2,3-dimethyl-2-butene, squalene or dibutyl phthalate. MCV is a useful technique for studying the mechanism of accelerated sulfur vulcanization. The effectiveness of this approach in studying the influence of activators on the course of the crosslinking reaction and the structure of the elastomer network has been confirmed by many researchers [48,49,50,51]. Moreover, the density functional study proposed by Maity and Pinĉák [52] could be helpful to explore the interfacial properties of the crosslinking system components. It is useful method to perform a systematic study of the adsorption energy of metal atom, e.g., zinc atom from vulcanization activator, with other curatives, e.g., sulfur.

### 3.2. The Effect of Vulcanization Activators on Tensile Properties and Hardness of SBR Vulcanizates

Having known that vulcanization activators and consequently reduction of zinc content affected the crosslink density of SBR vulcanizates, we then establish their tensile properties and hardness. These properties are known to be strongly dependent on the crosslink density of the vulcanizates [53,54]. The results are presented in Table 5.

The first parameter shown in Table 5 is the stress at a relative elongation of 300% (SE_300_), which depends on the crosslink density of the vulcanizate and increases with the number of crosslinks in the elastomer network. Replacing 5 phr of M-ZnO with 3 phr of N-ZnO did not have a significant influence on SE_300_ due to the similar crosslink density of the vulcanizates containing each of ZnO. Since reduced crosslink density, vulcanizates with zinc complexes, especially those containing methoxy groups, exhibited significantly lower SE_300_ compared to SBR cured with zinc oxides. The changes in the SE_300_ modulus of the vulcanizates strongly correlated with changes in their crosslink density. Thus, among the vulcanizates with zinc complexes, the highest SE_300_ was shown by that with HeBPP-Zn and the smallest by those with complexes containing methoxy groups.

**Figure 8:**
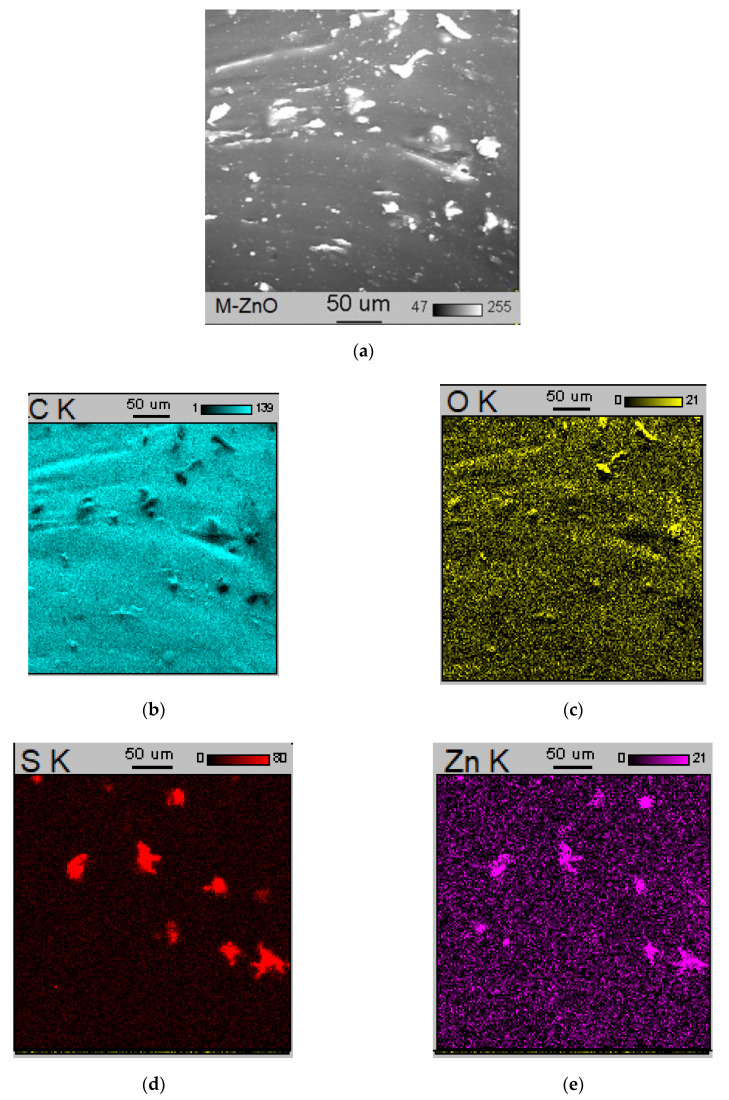
Scanning electron microscopy (SEM) image and Energy-dispersive X-ray spectroscopy (EDS) maps for SBR vulcanizate containing M-ZnO: (**a**) SEM image; (**b**) EDS map for C; (**c**) EDS map for O; (**d**) EDS map for S; (**e**) EDS map for Zn.

**Figure 9:**
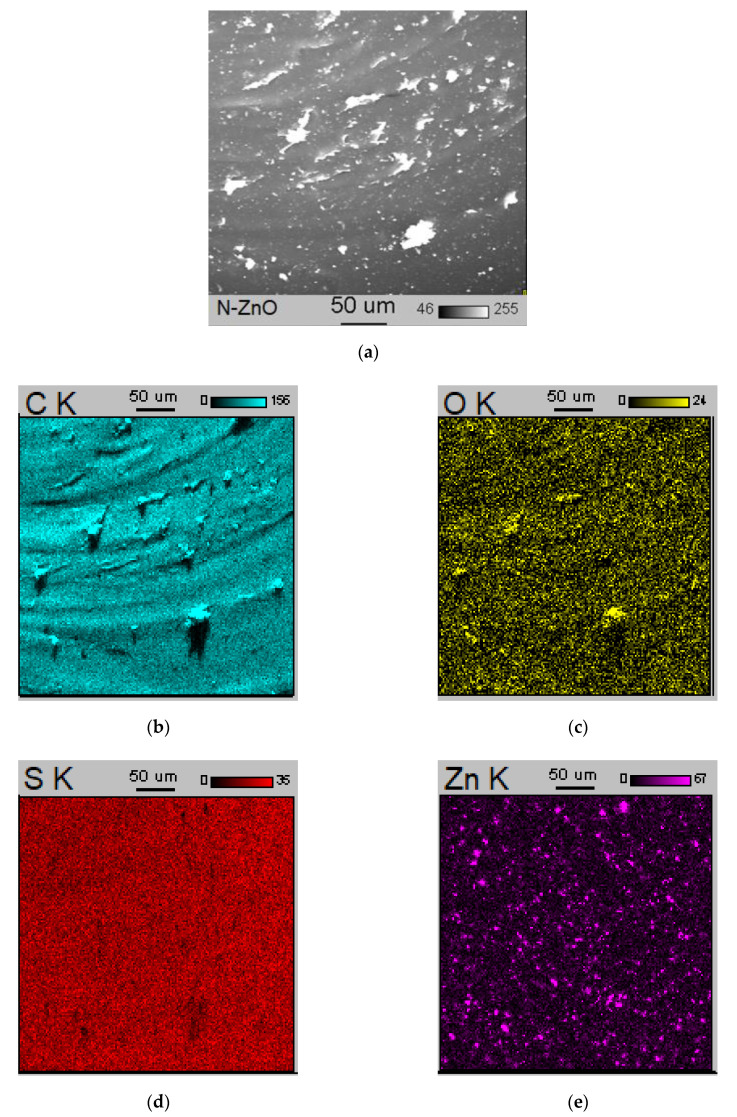
SEM image and EDS maps for SBR vulcanizate containing N-ZnO: (**a**) SEM image; (**b**) EDS map for C; (**c**) EDS map for O; (**d**) EDS map for S; (**e**) EDS map for Zn.

**Figure 10:**
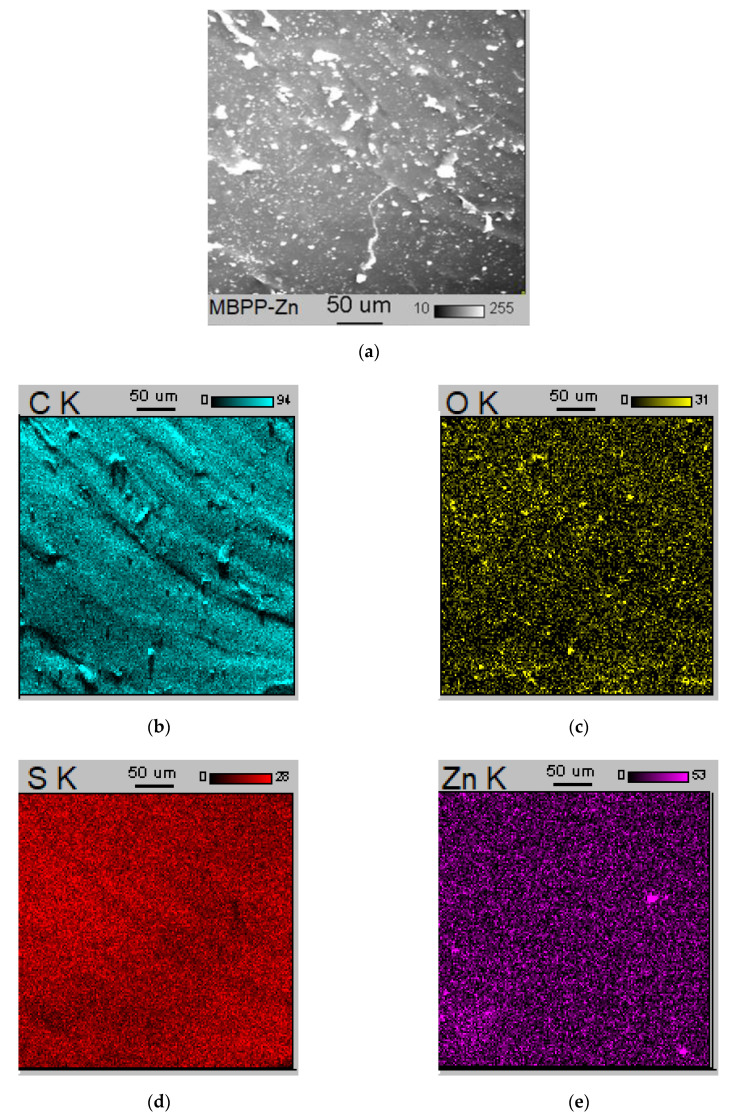
SEM image and EDS maps for SBR vulcanizate containing MBPP-Zn: (**a**) SEM image; (**b**) EDS map for C; (**c**) EDS map for S; (**d**) EDS map for Zn.

Similar to SE_300,_ the type of vulcanization activator had an impact on the tensile strength (TS) of SBR vulcanizates. The TS of the reference vulcanizate cured with M-ZnO was 14.1 MPa. Despite lower amount of zinc, application of N-ZnO enhanced the TS by approximately 2 MPa compared to the benchmark with M-ZnO. Similar TS was demonstrated by the vulcanizate containing a mixture of activators, i.e., N-ZnO and MBPP-Zn. Zinc complexes with 1,3-diketones improved the TS compared to SBR vulcanizates with M-ZnO and N-ZnO. The only exception was the vulcanizate cured with HeBPP-Zn, which exhibited TS similar to that containing N-ZnO. The highest TS of approximately 20 MPa was demonstrated by the vulcanizates with MBPP-Zn and tBuMBPP-Zn. Most importantly, despite lower content of zinc and consequently lower crosslink density, vulcanizates containing zinc complexes exhibited enhanced TS in comparison with SBR containing zinc oxides as activators. This could be due to the more homogeneous dispersion of the curatives in the crosslinked elastomer matrix. It is commonly known that the dispersion degree of both the filler particles and the crosslinking system particles, i.e., sulfur, vulcanization activators and accelerators, affects the mechanical properties of the vulcanizates [25]. Thus, SEM/EDS analysis was employed to study the dispersion of the filler and crosslinking system components in the SBR elastomer matrix. SEM images of the selected vulcanizates with EDS maps are presented in Figure 8, Figure 9 and Figure 10. The analysis was performed for the selected vulcanizates, i.e., those containing M-ZnO, N-ZnO or zinc complex MBPP-Zn.

An interesting observation was made by analyzing the SEM image and EDS maps of the vulcanizate containing 5 phr of M-ZnO (Figure 8). The EDS maps of Zn and S revealed that the microsized agglomerates, which could be seen in the SEM image, consisted of zinc sulfide (ZnS) formed as a by-product of the sulfur vulcanization. Thus, the use of 5 phr ZnO resulted in the formation of a significant amount of ZnS which showed the ability to agglomerate in the elastomer matrix. Consequently, deterioration in the mechanical properties of the vulcanizates was achieved since the agglomerates acted as stress concentration centers when the sample was subjected to external stress [32]. Carbon black (CB) used as a filler was quite homogeneously distributed in the elastomer matrix.

Analyzing SEM image of the vulcanizate containing 3 phr of N-ZnO (Figure 9), some microsized agglomerates heterogeneously dispersed in the elastomer matrix were observed, but EDS maps revealed that these agglomerates rather consisted of the CB particles, whereas Zn and S were quite uniformly distributed in the elastomer matrix and ZnS agglomeration did not occur. It was probably due to 40% lower content of N-ZnO compared to M-ZnO and consequently lower content of Zn, thanks to which less ZnS was formed during the vulcanization.

It should be noted that CB and curatives particles were the most homogeneously distributed in the SBR matrix crosslinked in the presence of zinc complex MBPP-Zn as an activator (Figure 10). Even though CB particles tended to agglomerate (Figure 10b), the resulting agglomerates were smaller in size and more uniformly dispersed in the elastomer matrix compared to the other vulcanizates. The EDS maps of S and Zn (Figure 10d,e) revealed a homogeneous dispersion of these components, and thus the vulcanization activator, in the elastomer. Due to the significantly lower content of Zn in the MBPP-Zn complex compared to M-ZnO, a very low amount of ZnS was formed during the vulcanization and thus, no agglomeration of ZnS in the elastomer matrix was seen. The homogeneous distribution of the solid additives in the SBR matrix crosslinked in the presence of MBPP-Zn resulted in the significantly increased tensile strength of the vulcanizate as compared to those containing M-ZnO or N-ZnO.

Using N-ZnO alternatively to commercially used M-ZnO did not have a meaningful effect on the vulcanizates flexibility. The reference vulcanizate with M-ZnO showed an elongation at break of 413%, whereas EB of the vulcanizates cured with N-ZnO was 458%. Owing to the lower number of crosslinks in the elastomer network, SBR cured with zinc complexes demonstrated higher EB, which was in the range of 507–572%. As expected, the highest EB manifested vulcanizates with the lowest crosslink density, i.e., those containing zinc complexes with methoxy groups.

N-ZnO and its mixture with MBPP-Zn did not significantly alter the hardness of the SBR vulcanizates compared with the reference sample cured with M-ZnO, which showed the hardness of 54 ShA. Vulcanizates with zinc complexes demonstrated the hardness in the range of 46–52 ShA. As expected, the lowest hardness was determined for the vulcanizates containing zinc complexes with methoxy groups due to the lowest crosslink density.

Most importantly, zinc complexes with 1,3-diketones, despite the approximately 90% lower content of zinc, allowed to obtain the vulcanizates with improved tensile strength, without significantly altering their flexibility and hardness compared to SBR elastomer cured with M-ZnO.

### 3.3. The Effect of Vulcanization Activators on Dynamic Mechanical Properties of SBR Vulcanizates

**Figure 11:**
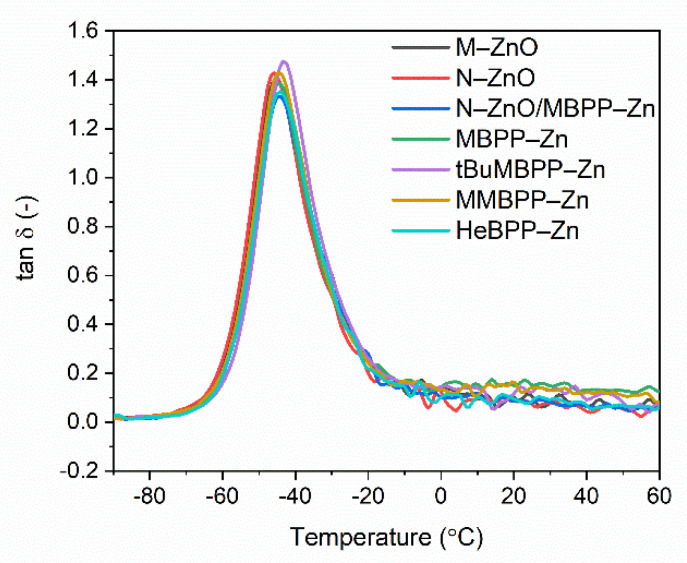
Loss factor (tan Δ) curves versus temperature of SBR vulcanizates containing ZnO and zinc complexes with 1,3-diketones as activators.

Elastomers often operate under conditions of variable deformation, and one of their main applications is vibration damping. Hence, apart from tensile properties, the mechanical performance of the vulcanizates under dynamic conditions is also important for potential applications of rubber products. Dynamic mechanical analysis (DMA) was employed to establish the influence of vulcanization activators with different content of zinc on the viscoelastic properties of SBR vulcanizates, and above all their ability to dampen vibrations. Thus, measurements of mechanical loss factor (tan Δ) as a function of temperature were performed. DMA curves of SBR vulcanizates containing different vulcanization activators are presented in Figure 11 and the results are summarized in Table 6.

The results of DMA analysis listed in Table 6 confirmed that applying N-ZnO alternatively to M-ZnO did not significantly alter the glass transition temperature (T_g_) of SBR elastomer, which was of approximately −45 °C. Considering the experimental error, zinc complexes had no meaningful influence on the T_g_ of SBR elastomers, which ranged from −43.4 to −44.7 °C.

Analyzing the height of the tan δ peak (tan δ_Tg_) on DMA curve, the values of tan δ at T_g_ were similar for most of the tested vulcanizates and varied within the range of measurement error. Thus, it was concluded that N-ZnO, MBPP-Zn and MMBPP-Zn did not affect the height of tan δ peak. On the other hand, vulcanizates with HeBPP-Zn and with a mixture of N-ZnO and MBPP-Zn exhibited only slightly lower tan δ_Tg_ compared to SBR cured with zinc oxides, so it is difficult to interpret this as a deterioration of the elastomer ability to dampen vibrations. Regarding the values of tan δ at 25 and 60 °C, i.e., in the elastic region, N-ZnO and its mixture with MBPP-Zn, as well as HeBPP-Zn had no meaningful influence on the damping properties of SBR elastomer. Furthermore, it was observed that vulcanizates containing zinc complexes with methoxy groups exhibited higher tan δ in the elastic region compared to other vulcanizates. Thus, they should demonstrate better damping properties in the elastic state compared to SBR cured with commercially used M-ZnO. Most importantly, despite significantly lower content of zinc in rubber compounds, applying N-ZnO and zinc complexes with 1,3-diketones alternatively to M-ZnO did not deteriorate the ability of SBR elastomer to dampen vibrations.

### 3.4. The Effect of Vulcanization Activators on Resistence of SBR Vulcanizates to Thermo-Oxidative Aging

**Figure 12:**
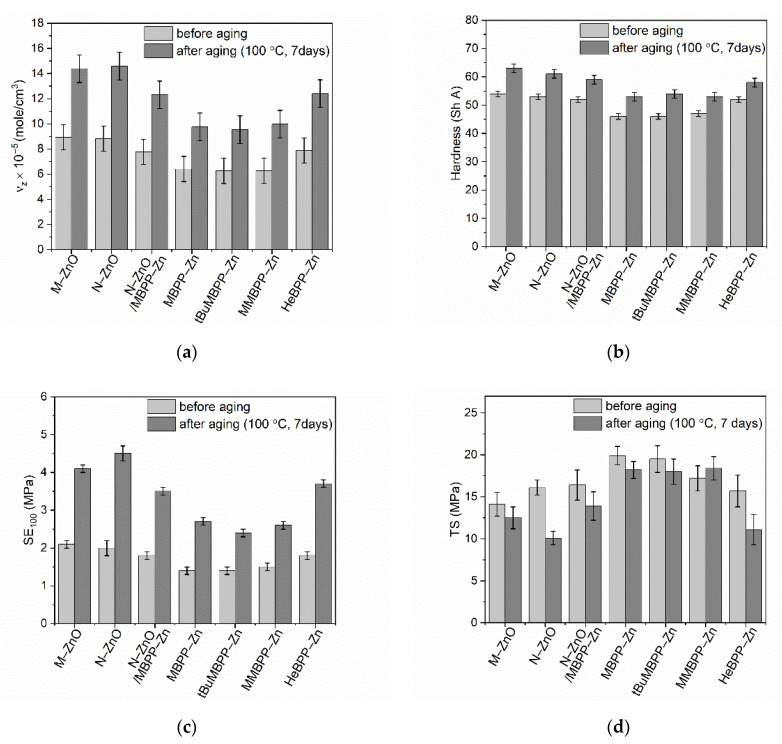

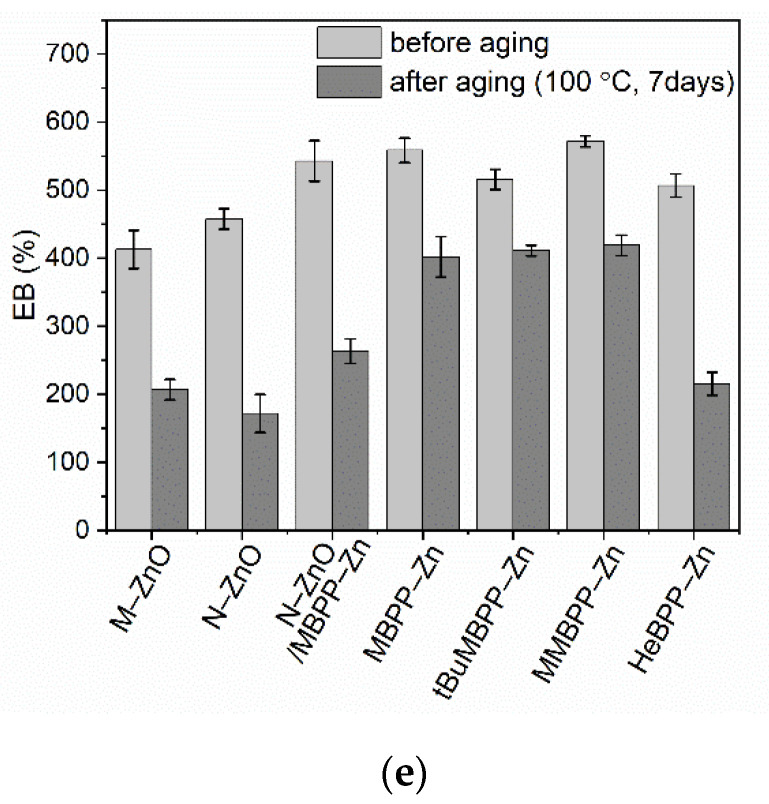
The influence of prolonged thermo-oxidation on the properties and crosslink density of SBR vulcanizates containing ZnO and zinc complexes with 1,3-diketones as activators: (**a**) crosslink density; (**b**) hardness; (**c**) stress at 100% relative elongation; (**d**) tensile strength; (**e**) elongation at break.

Rubber products are commonly used in outdoor applications; therefore, they are subject to long-term exposure to external factors causing their aging and, consequently, deterioration of functional properties. One of these factors is long-term thermo-oxidation. Therefore, the influence of alternative vulcanization activators, i.e., N-ZnO and zinc complexes with 1,3-diketones, on the resistance of SBR to thermo-oxidative aging was investigated. SBR vulcanizates were stored at 100 °C for 7 days, and then their crosslink density, mechanical performance and hardness were determined and compared with those of non-aged vulcanizates. The influence of thermo-oxidative aging on the properties of SBR vulcanizates is presented in Figure 12.

Prolonged exposure to thermo-oxidation significantly enhanced the crosslink density of SBR vulcanizates, especially those containing M-ZnO and N-ZnO as activators (Figure 12a). The increase in the crosslink density after thermo-oxidative aging is commonly reported for elastomers [55,56]. Slightly smallest changes in the crosslink density occurred for the vulcanizates containing a mixture of activators, i.e., N-ZnO and MBPP-Zn or HeBPP-Zn. In turn, the lowest increase in the crosslink density (significantly lower than for other vulcanizates) was observed for the vulcanizates with zinc complexes containing methoxy groups, which showed the lowest crosslink density before aging process. It probably resulted from the lower efficiency of zinc complexes having methoxy groups in activating vulcanization. It should be noted that vulcanization of elastomers, similar to most other technological processes, does not run with 100% efficiency. Vulcanization of rubber compounds at t_90_ as vulcanization time allows for the optimal use of the curatives under given conditions, which does not mean that they are completely consumed. Consequently, some unreacted crosslinkers remain inside the elastomer matrix and prolonged exposure to elevated temperature may initiate further crosslinking reactions resulting in additional crosslinks in the “aged” elastomer network. The results discussed earlier revealed that zinc complexes with methoxy groups were less active in vulcanization, therefore their ability to activate vulcanization during thermo-oxidative aging was also lower than other activators and hence a smaller increase in the crosslink density of the vulcanizates was achieved during aging. This assumption is also confirmed by the fact that the crosslink density of vulcanizates containing zinc oxides or the most active zinc complex, i.e., HeBPP-Zn, increased much more as a result of aging than that of SBR with less active zinc complexes.

Since hardness depends on the crosslink density, regardless of the vulcanization activator used, vulcanizates after thermo-oxidative aging exhibited by approximately 6–9 ShA higher hardness compared to the non-aged samples (Figure 12b). The highest hardness after thermo-oxidation was demonstrated by the vulcanizates containing M-ZnO (63 ShA) and N-ZnO (61 ShA), respectively.

Owing to the higher crosslink density, vulcanizates exhibited significantly higher modulus at 100% relative elongation (SE_100_) after aging process (Figure 12c). As expected, the highest enhancement of SE_100_ (by approximately 2 MPa) was demonstrated by the vulcanizates with the highest crosslink density, so those containing M-ZnO, N-ZnO and HeBPP-Zn. Vulcanizates with zinc complexes having methoxy groups demonstrated smaller changes in SE_100_ upon thermo-oxidation (reduction by 1.0–1.3 MPa compared to non-aged samples).

Regardless of the activator used, prolonged thermo-oxidation deteriorated the tensile strength (*TS*) of SBR vulcanizates. *TS* of the reference vulcanizate with M-ZnO was reduced by approximately 2 MPa (Figure 12d), whereas for N-ZnO containing vulcanizate significantly higher reduction in *TS* was achieved, i.e., 6 MPa. Regarding zinc complexes, the highest decrease in *TS* was demonstrated by the vulcanizate with HeBPP-Zn. On the other hand, vulcanizates containing zinc complexes with methoxy groups exhibited smaller changes in *TS* under thermo-oxidative aging, demonstrating *TS* by approximately 1.5–2.5 MPa lower compared to that of non-aged samples. Thus, it was concluded that SBR crosslinking during the thermo-oxidative aging caused this elastomer to be over-crosslinked, and consequently brittle and more susceptible to mechanical stress. Consequently, lower stress was sufficient to break the sample and its *TS* was reduced. The relationship between the *TS* and crosslink density of the elastomers is commonly known [2]. TS rises with the crosslink density of the elastomer to a certain critical value of the crosslink density, above which the elastomer becomes over-crosslinked. Further increasing the crosslink density of the elastomer causes the reduction in *TS*.

Thermo-oxidative aging significantly affected the elongation at break (*EB*) of SBR vulcanizates causing it to decrease compared to *EB* of the non-aged vulcanizates (Figure 12e). This resulted from the increase in the crosslink density under aging. As expected, the higher the increase in crosslink density of the vulcanizates, the greater the reduction in their elongation at break after thermo-oxidative aging was achieved. Thus, the highest reduction in *EB* by approximately 300% was observed for the vulcanizates containing N-ZnO and its mixture with MBPP-Zn or zinc complex HeBPP-Zn, whereas *EB* of the reference vulcanizate cured with M-ZnO was reduced by approximately 200% compared to that of the non-aged sample. Significantly smaller changes in *EB* after aging occurred for SBR containing zinc complexes with methoxy groups, for which *EB* was reduced by 100–150%.

It is difficult to estimate the resistance of an elastomeric material to aging processes by considering its individual mechanical parameters separately. Further, the aging coefficient *AF* was determined, which combines changes in individual parameters of the vulcanizates, i.e., *TS* and *EB* due to aging, and relates them to the values obtained for non-aged material. Results are presented in Table 7.

Referring to Equation (3) on the basis of which the aging coefficient (*AF*) was determined, the closer to 1 the value of *AF*, the greater the resistance of the material to aging, as this process causes less changes in the mechanical properties, i.e., *TS* and *EB*, of the vulcanizates.

The reference vulcanizate with M-ZnO demonstrated *AF* of approximately 0.4, so it was highly susceptible to thermo-oxidative aging. Applying N-ZnO and its mixture with MBPP-Zn did not affected the resistance of SBR to long-term thermo-oxidation (*AF* was in the range of measurement error). Similar *AF* was determined for the vulcanizate containing zinc complex HeBPP-Zn.

On the other hand, vulcanizates with zinc complexes containing methoxy groups exhibited significantly improved resistance to thermo-oxidative aging, demonstrating the *AF* in the range of 0.7–0.8. However, the improvement in aging resistance should not be attributed to the action of the zinc complexes themselves, but rather, as already mentioned, to their lower activity in the crosslinking process. Consequently, the increase in the crosslink density due to thermo-oxidative aging for vulcanizates containing zinc complexes with methoxy substituents was much lower than for other vulcanizates, resulting in less deterioration of their mechanical properties compared to vulcanizates with zinc oxides and HeBPP-Zn.

Most importantly, N-ZnO and zinc complexes with 1,3-diketones did not worsen the resistance of SBR to prolonged thermo-oxidation, which is important for their potential technological application as vulcanization activators alternative to M-ZnO, which enable the significant reduction in the amount of zinc in rubber products.

### 3.5. The Effect of Vulcanization Activators on Thermal Stability of SBR Vulcanizates

**Figure 13:**
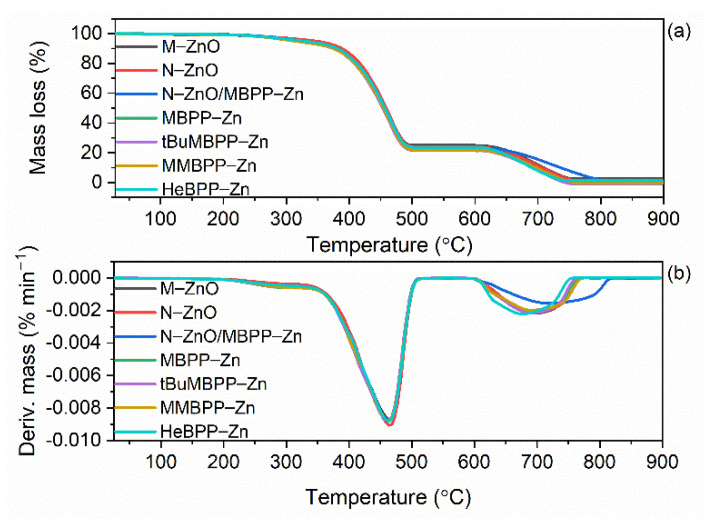
Thermogravimetric (TG) and Derivative Thermogravimetric (DTG) curves of SBR vulcanizates containing ZnO and zinc complexes with 1,3-diketones as activators: (**a**) TG curves; (**b**) DTG curves.

Zinc complexes with 1,3-diketones are organic compounds which are expected to decompose when heating to high temperatures. Thermal stability of elastomer composites depends on the thermal behavior of both elastomer matrix and components of the rubber compounds, especially the organic ones. Hence, the effect of zinc complexes on the thermal stability of SBR vulcanizates was investigated using thermogravimetry (TG). The results are shown in Figure 13 and summarized in Table 8.

Analyzing TG and DTG curves presented in Figure 13, two mass losses were achieved for the investigated vulcanizates. The first mass loss occurred in the temperature range of 25–600 °C and it was determined in argon atmosphere. Thus, it corresponds to the pyrolysis of elastomer matrix and organic ingredients such as vulcanization accelerators and zinc complexes with 1,3-diketones. Therefore, vulcanizates containing zinc complexes exhibited higher mass losses in the temperature range of 25–600 °C than the vulcanizates with zinc oxides (Table 8). In the temperature range of 600–900 °C argon was replaced by air, so the mass loss observed corresponds mainly to the combustion of carbon black, which was used as a filler, accompanied by the combustion of the residues from the first stage of thermal decomposition. Owing to the same content of filler, the mass loss in the temperature range of 600–900 °C was similar for all vulcanizates (approximately 22%) regardless of the applied vulcanization activator.

The mineral residue after thermal decomposition at 900 °C depended on the vulcanization activator used and it was the highest (approximately 4.2%) for the reference vulcanizate due to the content of 5 phr M-ZnO, which remained after thermal decomposition. The mineral residue at 900 °C for the vulcanizate with N-ZnO was of approximately 2.7% and resulted from 3 phr of N-ZnO. On the other hand, the residue after thermal decomposition of the vulcanizates with zinc complexes consisted of ash and was in the range of 0.2–0.5% because the organic zinc complexes underwent complete thermal decomposition under the conditions of measurement.

**Figure 14:**
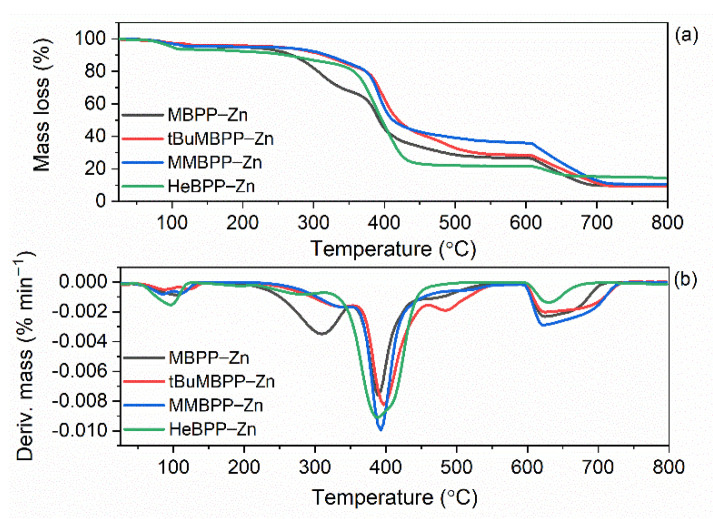
Thermal stability of zinc complexes with 1,3-diketones: (**a**) TG curves; (**b**) DTG curves.

The reference vulcanizate with M-ZnO began to thermally decompose at a temperature of approximately 334 °C. N-ZnO increased the onset decomposition temperature (T_5%_) of SBR to 346 °C. This may result from the network formed by ZnO nanoparticles dispersed in the elastomer matrix, which hinders the diffusion of gases and volatile products of thermal decomposition through the composite and thus prevents thermal decomposition [57]. As expected, zinc complexes with 1,3-diketones, especially those containing methoxy groups, deteriorated thermal stability of the vulcanizates. The T_5%_ temperature decreased by approximately 10–14 °C for the vulcanizates containing zinc complexes with methoxy groups and by 8 °C for HeBPP-Zn compared to the reference vulcanizate with M-ZnO. This resulted from the low thermal stability of zinc complexes compared to SBR elastomer matrix as confirmed by TG analysis of pure zinc complexes (Figure 14, Table 9). Zinc complexes with 1,3-diketones demonstrated different thermal stability depending on their structure and were characterized by T_5%_ in the range of 141–249 °C, whereas T_DTG_ ranged from 404 to 413 °C for HeBPP-Zn and tBuMBPP-Zn, respectively. The most thermally stable zinc complex was tBuMBPP-Zn, while the lowest T_5%_ was determined for HeBPP-Zn probably due to the fragmentation of long heptyl chain, which initiated thermal decomposition of this complex. The thermal decomposition of aliphatic-aromatic compounds with long alkyl substituents was reported to begin with the fragmentation of long alkyl chains [58]. Deterioration of the thermal stability of SBR elastomer by incorporation of organic additives, which decompose at lower temperatures compared to pure SBR was confirmed by Prochon et al. [59].

On the other hand, vulcanization activators did not significantly affect the peak temperature of DTG curve (T_DTG_) which was in the range of 480–483 °C. Therefore, it was concluded that decomposition of SBR vulcanizates with zinc complexes began at lower temperatures but proceeded at a rate similar to that of the ZnO-containing vulcanizates. Most importantly, regardless of the vulcanization activator applied, SBR vulcanizates were thermally stable up to a temperature of approximately 310 °C, which is sufficient for their technological applications.

## 4. Conclusions

The possibility of zinc amount reduction in SBR composites filled with carbon black was investigated. For this purpose, N-ZnO and complexes of zinc with 1,3-diketones were applied as vulcanization activators alternatively to the commercially used M-ZnO.

Applying N-ZnO enabled to reduce the content of zinc by 40% compared to M-ZnO without detrimental influence on the cure characteristic of SBR compounds and crosslink density of the vulcanizates. N-ZnO did not significantly affect the resistance of SBR vulcanizates to thermo-oxidative aging and their ability to dampen vibrations. Moreover, vulcanizates with N-ZnO exhibited enhanced tensile strength and thermal stability compared to SBR cured with M-ZnO.

Despite the 80–90% lower zinc content, zinc complexes with 1,3-diketones effectively activated the vulcanization of SBR compounds and their activity depended on the zinc complex structure, i.e., the availability of zinc ions to react with curatives. The highest activity in vulcanization was demonstrated by the zinc complex with two phenyl rings and heptyl chains, i.e., HeBPP-Zn, in which the attainability of zinc was significantly better compared to zinc complexes with diketones having four phenyl rings, methoxy groups and t-butyl chains. Thus, HeBPP-Zn had no significant influence on the time and temperature of rubber compounds vulcanization. Compared to SBR cured with M-ZnO, vulcanizate with HeBPP-Zn exhibited slightly lower crosslink density, slightly worse damping properties and thermal stability, similar resistance to thermo-oxidative aging and improved tensile strength. Due to the steric hindrance, which hindered the availability of zinc to react with curatives, zinc complexes with four phenyl ring and methoxy groups showed lower activity in the vulcanization, and consequently vulcanizates with these complexes demonstrated lower crosslink density and hardness. However, it should be noted that despite lower content of crosslinks, vulcanizates with MBPP-Zn, tBuMBPP-Zn and MMBPP-Zn exhibited significantly enhanced tensile strength (of approximately 20 MPa) and resistance to thermo-oxidative aging, as well as improved damping properties compared to SBR cured with M-ZnO. In addition, regardless of the vulcanization activators, SBR composites were thermally stable up to a temperature of 310 °C.

Most importantly, performed studies confirmed that zinc complexes with 1,3-diketones can be successfully used as vulcanization activators which allow a 80–90% reduction in the content of zinc compared to SBR compounds with commercially used M-ZnO. This is important for ecological reasons. Moreover, the lower content of zinc in rubber compounds the lower amount of ZnS is formed as a by-product of crosslinking reactions. ZnS is commonly known to stain the vulcanization molds, which makes it necessary to clean them after the vulcanization cycle is completed. Thus, the reduction of zinc is important also for technological reasons.

## Figures and Tables

**Table 1:** General formulations of the styrene-butadiene-rubber (SBR) compounds, parts per hundred of rubber (phr); MBT, 2-mercaptobenzothizole; CBS, N-cyclohexyl-2-benzothiazolesulfenamide; M-ZnO, microsized zinc oxide; N-ZnO, zinc oxide nanopowder; CB, carbon black.

Ingredient	Reference Sample	SBR Compound with N-ZnO	SBR Compound with Zinc Complexes ^1^
SBR	100	100	100
Sulfur	2	2	2
CBS	1	1	1
MBT	1	1	1
CB	30	30	30
M-ZnO	5	-	-
N-ZnO	-	3	-
Zinc complex	-	30	3

^1^ One of the rubber compounds contained a mixture of N-ZnO (1.5 phr) and MBPP-Zn (1.5 phr).

**Table 2:** Amount of zinc in SBR compounds containing different vulcanization activators.

Activator	Amount of Zinc in Activator (mmolg)	Amount of Zinc in SBR Compound (mmol/100 g of SBR)
M-ZnO	12.3	61.5
N-ZnO	12.3	36.9
MBPP-Zn	1.7	5.1
tBuMBPP-Zn	2.2	6.6
MMBPP-Zn	1.6	4.8
HeBPP-Zn	1.8	5.4

**Table 3:** Cure characteristics at 160 °C and crosslink density of SBR composites containing ZnO and zinc complexes with 1,3-diketones as activators (*S_min_*, minimum torque; ∆*S*, torque increase; t_02_, scorch time; t_90_, optimal vulcanization time; ν**_t_**, crosslink density; SD: *S_min_* ± 0.1 MPa; ∆*S* ± 2.0 MPa; t_02_ ± 0.1 min.; t_90_ ± 0.3 min.; ν_t_ ± 0.2 × 10^−5^ mole/cm^3^).

SBR Compounds	*S_min_* (dNm)	Δ*S* (dNm)	t_02_ (min)	t_90_ (min)	ν_t_·10^−5^ (mole/cm^3^)
M-ZnO	1.1	16.0	1.0	4.7	8.9
N-ZnO	1.1	15.8	1.4	4.8	8.8
N-ZnO/MBPP-Zn	1.1	13.5	1.1	3.7	7.8
MBPP-Zn	1.0	9.9	0.6	2.0	6.4
tBuMBPP-Zn	1.0	9.6	0.6	1.8	6.3
MMBPP-Zn	1.0	9.8	0.6	2.0	6.3
HeBPP-Zn	1.1	13.3	1.3	4.3	7.9

**Table 4:** Temperature and enthalpy of curing determined by differential scanning calorimetry (DSC) for SBR compounds containing ZnO and zinc complexes with 1,3-diketones as activators (T_cur_, curing temperature; ∆H, curing enthalpy; T_g_, glass transition temperature; SD: temperature ± 9.0 °C; ΔH ± 2.6 J/g; T_g_ ± 1 °C).

SBR Compounds	T_cur_ (°C)	∆H (J/g)	T_g_ (°C)
M-ZnO	163–193	7.2	−51.7
N-ZnO	165–195	6.8	−51.8
N-ZnO/MBPP-Zn	168–206	8.4	−51.6
MBPP-Zn	142–177	5.9	−51.5
tBuMBPP-Zn	144–178	5.6	−51.2
MMBPP-Zn	141–177	5.4	−51.9
HeBPP-Zn	166–206	6.4	−51.7

**Table 5:** Tensile properties and hardness of SBR compounds containing ZnO and zinc complexes with 1,3-diketones as activators.

SBR Vulcanizate	SE_300_ (MPa)	TS (MPa)	EB (%)	H (ShA)
M-ZnO	9.0 ± 0.1	14.1 ± 1.4	413 ± 28	54 ± 1
N-ZnO	8.6 ± 0.2	16.1 ± 0.9	458 ± 15	53 ± 1
N-ZnO/MBPP-Zn	7.0 ± 0.1	16.4 ± 1.8	543 ± 30	52 ± 1
MBPP-Zn	5.4 ± 0.1	19.9 ± 1.1	559 ± 18	46 ± 1
tBuMBPP-Zn	5.4 ± 0.1	19.5 ± 1.6	516 ± 15	46 ± 1
MMBPP-Zn	5.5 ± 0.1	17.2 ± 1.5	572 ± 8	47 ± 1
HeBPP-Zn	7.3 ± 0.1	15.7 ± 1.9	507 ± 17	52 ± 1

**Table 6:** Glass transition temperature (T_g_) determined by dynamic mechanical analysis (DMA) and mechanical loss factor (tan δ) of SBR vulcanizates containing ZnO and zinc complexes with 1,3-diketones as activators (SD: T_g_ ± 1.3 °C; tan δ_Tg_ ± 0.3; tan δ_25–60 °C_ ± 0.01).

SBR Vulcanizates	T_g_ (°C)	tan δ_Tg_ (−)	tan δ_25°C_ (−)	tan δ_60°C_ (−)
M-ZnO	−45.5	1.40	0.10	0.07
N-ZnO	−45.8	1.43	0.08	0.06
N-ZnO/MBPP-Zn	−44.2	1.33	0.08	0.08
MBPP-Zn	−44.2	1.39	0.15	0.12
tBuMBPP-Zn	−43.4	1.48	0.13	0.10
MMBPP-Zn	−44.7	1.43	0.14	0.10
HeBPP-Zn	−44.1	1.35	0.09	0.07

**Table 7:** Thermo-oxidative aging coefficient (*AF*) of SBR vulcanizates containing ZnO and zinc complexes with 1,3-diketones as activators (SD: *AF* ± 0.1).

SBR Vulcanizate	*AF* (−)
M-ZnO	0.4
N-ZnO	0.3
N-ZnO/MBPP-Zn	0.4
MBPP-Zn	0.7
tBuMBPP-Zn	0.7
MMBPP-Zn	0.8
HeBPP-Zn	0.3

**Table 8:** Onset temperature of thermal decomposition (T_5%_), DTG peak temperature (T_DTG_) and total mass loss (∆m) during decomposition of SBR compounds containing ZnO and zinc complexes with 1,3-diketones as activators (SD: T_5%_ ± 1.1 °C; T_DTG_ ± 1.0 °C; ∆m ± 1.6%).

SBR Vulcanizate	T_5%_ (°C)	T_DTG_ (°C)	Δm_(25–600 °C)_ (%)	Δm_(600–900 °C)_ (%)	Residue at 900 °C (%)
M-ZnO	334	482	73.5	22.3	4.2
N-ZnO	346	483	74.6	22.7	2.7
N-ZnO/MBPP-Zn	328	480	76.4	22.3	1.3
MBPP-Zn	310	481	77.5	22.2	0.3
tBuMBPP-Zn	312	480	77.3	22.2	0.5
MMBPP-Zn	313	481	77.7	22.1	0.2
HeBPP-Zn	320	481	77.4	22.2	0.4

**Table 9:** Thermal stability of zinc complexes with 1,3-diketones (T_5%_, decomposition temperature at 5% of the mass change; T_DTG_, DTG peak temperature; standard deviations (SD): T_5%_, T_DTG_ ± 2 °C).

Zinc Complex	T_5%_ (°C)	T_DTG_ (°C)
MBPP-Zn	189	405
tBuMBPP-Zn	249	413
MMBPP-Zn	225	408
HeBPP-Zn	141	404

## Data Availability

The data presented in this study are available on request from the corresponding author.
